# Assimilation of Diazotrophic Nitrogen into Pelagic Food Webs

**DOI:** 10.1371/journal.pone.0067588

**Published:** 2013-06-28

**Authors:** Ryan J. Woodland, Daryl P. Holland, John Beardall, Jonathan Smith, Todd Scicluna, Perran L. M. Cook

**Affiliations:** 1 Water Studies Centre, School of Chemistry, Monash University, Clayton, Victoria, Australia; 2 School of Biological Sciences, Monash University, Clayton, Victoria, Australia; 3 South East Algae Project SEAPRO, Metung, Victoria, Australia; Dowling College, United States of America

## Abstract

The fate of diazotrophic nitrogen (N_D_) fixed by planktonic cyanobacteria in pelagic food webs remains unresolved, particularly for toxic cyanophytes that are selectively avoided by most herbivorous zooplankton. Current theory suggests that N_D_ fixed during cyanobacterial blooms can enter planktonic food webs contemporaneously with peak bloom biomass via direct grazing of zooplankton on cyanobacteria or via the uptake of bioavailable N_D_ (exuded from viable cyanobacterial cells) by palatable phytoplankton or microbial consortia. Alternatively, N_D_ can enter planktonic food webs post-bloom following the remineralization of bloom detritus. Although the relative contribution of these processes to planktonic nutrient cycles is unknown, we hypothesized that assimilation of bioavailable N_D_ (e.g., nitrate, ammonium) by palatable phytoplankton and subsequent grazing by zooplankton (either during or after the cyanobacterial bloom) would be the primary pathway by which N_D_ was incorporated into the planktonic food web. Instead, *in situ* stable isotope measurements and grazing experiments clearly documented that the assimilation of N_D_ by zooplankton outpaced assimilation by palatable phytoplankton during a bloom of toxic *Nodularia spumigena* Mertens. We identified two distinct temporal phases in the trophic transfer of N_D_ from *N. spumigena* to the plankton community. The first phase was a highly dynamic transfer of N_D_ to zooplankton with rates that covaried with bloom biomass while bypassing other phytoplankton taxa; a trophic transfer that we infer was routed through bloom-associated bacteria. The second phase was a slowly accelerating assimilation of the dissolved-N_D_ pool by phytoplankton that was decoupled from contemporaneous variability in *N. spumigena* concentrations. These findings provide empirical evidence that N_D_ can be assimilated and transferred rapidly throughout natural plankton communities and yield insights into the specific processes underlying the propagation of N_D_ through pelagic food webs.

## Introduction

Eutrophication is recognized as a critical contributing factor to systemic ecosystem shifts in shallow coastal habitats [1], [2] and despite a growing understanding of the proximate symptoms of eutrophication (e.g., hypoxia, reduced benthic-pelagic coupling, species loss), the ultimate consequences for biogeochemical processes and ecological function remain relatively unknown [3], [4]. Due to their potentially rapid growth rates and direct physiological response to nutrient conditions, noxious blooms of phytoplankton are one of the most common and potentially devastating symptoms of cultural eutrophication in coastal ecosystems [5]. Planktonic cyanobacteria are particularly problematic because they are often toxic and form light-limiting surface scums. Blooms of diazotrophic cyanobacteria are unique in that diazotrophy (the ability to fix dissolved N_2_) circumvents nitrogen limitation [6], [7] while generating an input of novel nitrogen to aquatic food webs [6], [7], [8].

Herbivorous grazing by microzooplankton (c. 20–200 µm body length) and mesozooplankton (c. 200–2000 µm) is the primary process by which phytoplankton are consumed and organic nutrients remineralized; yet, toxic cyanobacteria are often avoided by planktonic herbivores [9], [10], [11]. Despite this, grazing by zooplankton on cyanobacteria has been hypothesized to explain the rapid assimilation of diazotrophic nitrogen (N_D_) from a toxic cyanobacterium into zooplankton [12]. In the absence of direct grazing, cyanobacterial cells are assumed to accumulate throughout the bloom period until microbial remineralization of detrital bloom material renders the N_D_ available to other, more palatable phytoplankton groups [13], and thence to zooplankton. Alternatively, it has been shown that viable cyanobacterial cells are capable of releasing up to 35% of fixed N_2_ to surrounding microenvironments as inorganic NH_4_^+^ [14], [15] in addition to releasing dissolved organic nitrogen (DON) exudates [16]. This extracellular release of dissolved inorganic nitrogen (DIN) and DON to surrounding waters represents a lateral subsidy of N_D_ to the consortial communities of bacteria that colonize cyanobacterial cells [9], [17] and to sympatric phytoplankton. Stimulation of bacterial communities and palatable phytoplankton taxa through extracellular exudation of N_D_ represents an indirect trophic pathway by which fixed N_2_ in ungrazed cyanobacteria can be assimilated into planktonic food webs.

Our current understanding of plankton trophodynamics and cyanobacterial physiology suggests that N_D_ derived from cyanobacteria can enter planktonic food webs contemporaneously with peak bloom biomass via: 1) direct grazing of zooplankton on cyanobacteria [9]; or 2) zooplankton foraging on either palatable phytoplankton or consortial bacteria that have assimilated nitrogenous exudates from viable cyanobacterial cells [12], [18]. Alternatively, the transfer of N_D_ to planktonic food webs can occur post-bloom following the coupled respiration and remineralization of particulate and dissolved bloom biomass [13]. We tested this conceptual model during a bloom of the cyanobacterium *Nodularia spumigena* in Lake King, Gippsland Lakes, Australia. The filamentous *N. spumigena* is a toxic diazotrophic cyanobacterium that produces the hepatotoxin nodularin [9] and there is substantial evidence that herbivorous zooplankton preferentially avoid grazing on this species under natural conditions [9], [11], [19]. We hypothesised that palatable phytoplankton taxa would be central to the trophic transfer of N_D_, be it via uptake of N_D_ exuded by *N. spumigena* during the bloom or remineralised post bloom. We expected evidence of a bloom-contemporary transfer of N_D_ to zooplankton, although we hypothesized that this was likely to be small relative to post-bloom processes. We also hypothesized that direct grazing of zooplankton on *N. spumigena* would be negligible and we conducted grazing experiments to quantify the role of direct grazing. We used δ^15^N stable isotope data to track the fate of bloom-derived N_D_ and generate estimates of mass-specific flux rates of N_D_ through different functional groups of the plankton community. Patterns in N_D_ flux rates and compositional changes in the plankton community were interpreted to infer the role of contemporaneous (i.e., bacterially-mediated lateral trophic transfer) and post-bloom processes (i.e., cellular decomposition) as delivery mechanisms of N_D_ to the planktonic food web. Our study verifies the transfer of N_D_ from cyanobacteria to multiple elements of the estuarine food web and identifies the specific trophic routing that facilitates the propagation of N_D_ to higher trophic levels.

## Methods

The Gippsland Lakes are an interconnected chain of three lagoonal estuaries in southeastern Victoria (Australia) that are subject to anthropogenic eutrophication and experience episodic blooms of *Nodularia spumigena* Mertens [8]. In anticipation of a bloom during the 2011–2012 austral summer, plankton sampling was initiated at two open-water sampling sites in Lake King (north site: 37.8757° S, 147.7574°E; south site: 37.9161° S, 147.7792°E). No special permitting was required because sampling did not occur within a protected area and was limited to the collection of ambient water and planktonic organisms. Water and plankton samples were collected from each sampling location on 21 September, 5 October, 28 November 2011, and at weekly–biweekly intervals from 15 December 2011 to 28 March 2012. Hereafter, calendar days will be reported as study days, with study day 1 = 1 September 2011.

### Grazing Experiments

Bioassay experiments were conducted using the dilution method [20] to quantify growth rate and grazing pressure on different components of the autotrophic community. For each iteration of the bioassays (N = 9), ambient water samples were collected from the northern sampling site in opaque 5 L containers for transfer back to the laboratory. Here and in later descriptions, all samples were collected from c. 20 to 25 cm subsurface to avoid potential biases associated with the unique composition of matter in surface scums.

Three replicate 100-ml incubations were prepared in 150 ml chambers by diluting ambient estuary water with 0.7 µm GF/F filtered water in ratios of 1∶0 (i.e., ambient), 1∶4 and 1∶19. Incubation chambers were maintained in a water bath at *in situ* temperatures under natural light conditions: ∼ 100 µmol photons m^−2^ s^−1^ illumination and a 14-hr light:10-hr dark cycle. The composition of the autotrophic community was analysed non-destructively in a Phytopam (Heinz Walz GmbH, Effeltrich, Germany), which quantifies the relative contribution of green (chlorophytes), brown (diatoms and dinoflagellates) and cyan (cyanobacteria) groups to the total measured chlorophyll fluorescence (factory calibration used). Regression of Phytopam output against direct cell counts showed the intensity of the cyan channel to be an excellent predictor of *N. spumigena* biovolume (n = 16, *r^2^* = 0.95, *N. spumigena* [mm^−3^ L^−1^] = 1.53×Intensity_cyan_ –0.36). During the experiments, there was evidence that *N. spumigena* fluorescence was contributing to the intensity of the green channel; therefore, the green channel was not considered in the data analysis. This was justified on the basis that chlorophytes made a negligible contribution to total phytoplankton biomass throughout this study. Dark-adapted fluorescence measurements were taken at the start of each incubation and every 1–2 days thereafter from each chamber. Incubations were maintained for 7–12 d; however, growth and grazing rate calculations were based on the initial response of each phytoplankton group (i.e., until growth stabilized or reversed) to minimize the influence of bottle-effects on rate measurements.

Daily growth rates of phytoplankton (*G*, d^−1^) associated with color channels were calculated for each replicate incubation chamber. A least-squares linear regression was fitted to *G* (dependent variable) across the three different dilution regimes (independent variable) and the extrapolated intercept interpreted as the maximum predicted daily growth rate (*G*_max_) in the absence of zooplankton grazing. The difference between *G*_max_ and the mean *G* observed in the undiluted control samples yields an estimate of grazing rate (*r*_G_, d^−1^) by zooplankton on each phytoplankton group. Differences in *r*_G_ between the cyan and brown groups were tested with a separate variance *t*-test due to evidence of heteroskedasticity in the data (variance ratio test; *F*_17, 23_ = 0.05, *p*<0.001).

### Sampling the Plankton Community

We targeted four unique components of the planktonic food web for stable isotope analysis: phytoplankton (0.7–63 µm), microzooplankton (80–149 µm), mesozooplankton (≥150 µm), and *N. spumigena* isolates. At each sampling site, an ambient 2 L water sample was collected and immediately preserved with Lugol’s iodine solution to provide an unfiltered sample for later identification and quantification of the plankton community. Identification and direct counts of plankton taxa were conducted by an experienced plankton ecologist using light microscopy and replicate counts with a haemocytometer (small cells only <3 µm) and Sedgewick Rafter chamber (larger taxa). Biovolumes of *N. spumigena* were estimated using the Sedgewick Rafter chamber on the Lugol’s preserved samples. Additional biovolume estimates were provided by ancillary sampling conducted by the Victorian Environmental Protection Agency (EPA).

The 0.7–63 µm phytoplankton samples were collected by filtering a known volume of 63 µm prefiltered ambient water onto pre-combusted glass fiber filters (Whatman GF/F, 0.7 µm nominal pore dia.). Prefiltering removed most *N. spumigena* filaments in the sample and subsequent analysis verified that the day-specific *δ* ^15^N of the 63 µm filtered phytoplankton was significantly enriched relative to ambient water samples during the bloom (one-tailed paired *t*-test; *t* = 2.08, *df* = 7, *p* = 0.04) but not after (one-tailed paired *t*-test; *t* = 0.08; *df* = 7, *p* = 0.47). Larger size classes of plankton were collected by towing an 80 µm plankton net for 3–5 min at ∼ 3 km h^−1^ at each location. High concentrations of *N. spumigena* filaments in net samples precluded the partitioning of plankton size classes in the field; therefore, whole plankton samples were stored in sample bottles with 0.7 µm filtered ambient water and held on ice for up to several hours until stored at −20°C in the laboratory.

In the laboratory, samples were thawed and a pipette used to siphon the positively buoyant *N. spumigena* filaments from other plankton in the water samples. A 150-µm sieve was used to separate the remaining plankton into the 80–149 µm microzooplankton and ≥150 µm mesozooplankton size classes. After filtering, light microscopy was used to verify that biomass of *N. spumigena* was a negligible component of the size-fractioned plankton samples. If substantial *N. spumigena* filaments were apparent (i.e., more than ∼ 10% numerical abundance), size-fractioned samples were diluted with deionized water and centrifuged at 2000–4000 rpm in a refrigerated swinging bucket centrifuge for 5–10 min. Non-*N. spumigena* plankton typically settled out first and formed the apex of the sediment; whereas, *N. spumigena* remained suspended in solution or formed a recognizable green upper layer on the sediment. The supernatant and any sedimented *N. spumigena* was discarded and the remaining sediment re-examined under a microscope. This procedure was repeated until the bulk of the sample biomass was composed of non-*N. spumigena* taxa. The sample material was then refiltered and triple-rinsed with deionized water to remove any particulates generated during the centrifugation process.

The size classes in this study generally correspond to taxonomically and functionally different plankton components [12], [21] and visual examination of a subset of our size-fractionated samples supported our broad classifications. Biomass in the phytoplankton samples was primarily autotrophic phytoplankton (e.g., diatoms: *Aulacoseira* sp., *Pseudo-nitzschia* sp., *Skeletonema costatum*), with scattered mixotrophs (e.g., dinoflagellates: *Ceratium furca*, *Prorocentrum minimum*) and heterotrophs (e.g., ciliates: *Favella* sp.) also present at low levels. Microzooplankton biomass was primarily heterotrophic zooplankton (e.g., copepod nauplii, ciliates) with mixotrophic dinoflagellates and *Coscinodiscus* sp. contributing a small fraction to the total organic biomass. Biomass in the mesozooplankton was almost exclusively heterotrophic zooplankton (e.g., copepods: *Oncaea media*, *Sulcanus conflictus*; bivalve veligers).

Samples were held in an oven at 60°C until completely dried (min 48 hrs), after which they were ground to a fine homogenous powder and packed into sterile tin capsules for N-isotope analysis. Isotope samples were analysed on a Hydra 20–22 isotope ratio mass-spectrometer and coupled ANCA-GSL2 elemental analyser (Sercon Ltd., UK) at Monash University. Isotope values are reported in the *δ*-notation relative to ambient air with precision = 0.1‰.

Least-squares linear regression was used to assess the magnitude of observed declines in *δ* ^15^N of plankton groups at each site from initial conditions (pre-bloom) to the termination of the bloom (22 September 2011–7 February 2012).

### Flux Rate Calculations

Daily uptake rates of diazotrophic N (N_D_) into the non-*N. spumigena* plankton groups were calculated using isotope mass balances. Mass-specific rate calculations were carried out using the mass-balance approach described by Montoya et al. [22], modified to include a trophic fractionation term [23], [24]. Trophic fractionation of *δ* ^15^N was included in estimates of N_D_ flux into the micro- and mesozooplankton groups, but not the phytoplankton group. The proportion of N_D_ present in the biomass of plankton group *i* at day *t* was estimated by calculating the proportion of N_D_ in each plankton sample, *p*, using a simple two-endmember mixing model:
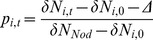
(1)where *δN_i,t_* is the nitrogen isotope value of plankton group *i* at time *t*, *δN_i,0_* is the nitrogen isotope value of the plankton group prior to the *N. spumigena* bloom, *δN_Nod_* is the average nitrogen isotope value of *N. spumigena*, and *Δ* is the trophic fractionation value assigned to that particular plankton group.

The specific rate of nitrogen uptake, *V* (d^−1^), was calculated as:
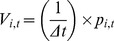
(2)where *Δt* is the interval in days between samples and *p_i,t_* is the proportion of the total nitrogen derived from N_D_ in plankton group *i* at day = *t*. Mass-specific uptake of N_D_, *ρ_i,t_* (nmol N mg^−1^ d^−1^), was then calculated as:
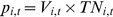
(3)where TNi,t is the concentration of total N in plankton group i at day = t given as nmol N per mg dry weight.

We used a nominal one-trophic level enrichment of *Δ* = 3.4 ‰ [24], [25] for the zooplankton size classes relative to their primary putative N-source (phytoplankton). During initial model construction, sensitivity analysis showed that *ρ* estimates increased by 14.9±8.2 (SD) nmol N mg^−1^ d^−1^ (microzooplankton) and 27.8±13.0 nmol N mg^−1^ d^−1^ (mesozooplankton) for each 1 ‰ increase in the value of the *Δ* parameter. These patterns were consistent: estimates of N_D_ flux increased (decreased) for a plankton group if the group was assigned to a higher (lower) estimated trophic position or if the value of the fractionation term was increased (decreased). Although the absolute value of our flux estimates are contingent on the value of *Δ*, the relative changes and the overall patterns among plankton groups were unaffected by the presence or absence of the fractionation term. In the absence of empirical trophic position estimates for zooplankton in this study and based on the flexible feeding ecologies of these taxa, we opted to include *Δ* while using a conservative estimate of 1 trophic exchange between zooplankton groups and their putative food source.

Fractionation of *δ* ^15^N_D_ can also occur during assimilation of dissolved N_D_ by phytoplankton [26], [27] or nitrification of diazotrophic NH_4_^+^ in the water column [28], [29]. Isotopic fractionation during these processes was not considered here due to persistently low DIN concentrations in the environment and evidence of N-limitation among phytoplankton. Total DIN concentrations in the upper water column were consistently low throughout the bloom (north = 0.89±0.53 [SD] µM; south = 1.19±0.95 µM) suggesting N-limiting conditions among non-diazotrophic phytoplankton. Indeed, ancillary nutrient additions conducted in tandem with grazing experiments indicated marked N-limitation among diatoms and dinoflagellates throughout the study period (See Appendix S1). In addition, phytoplankton will assimilate both NH_4_^+^ and NO_3_^−^, particularly when N-limited; thus, the mass-balance of the combined uptake of total DIN by phytoplankton would not reflect the kinetic discrimination of ^15^N_D_ during nitrification. Taken together, these conditions suggest that declines in *δ* ^15^N values across plankton groups were primarily driven by assimilation of N_D_ into their tissues rather than a reduction in the concentration of ^15^N in the DIN pool.

The focus of this study is on the flux of N_D_ through planktonic food webs; yet ultimately, the planktonic web is only one of several interacting components of the larger aquatic food web. We investigated the transfer of bloom-associated N_D_ to the demersal food web by analyzing the white muscle and liver tissue *δ* ^15^N composition of a commercially caught finfish, black bream *Acanthopagrus butcheri*, collected over the course of the study. The *δ* ^15^N composition of fish muscle and liver tissue equilibrate to the isotopic composition of diet (with fractionation) at different rates [30]; therefore, differential shifts in the *δ* ^15^N value of these tissues during a time-series can provide an indicator of the timing and relative magnitude of N_D_ flux from the pelagic food web to the demersal food web. All fish were collected with gill nets from areas affected by the *N. spumigena* bloom and immediately frozen. White muscle from the dorsal musculature and liver tissue were dried at 60°C, then pulverized and analysed for *δ* ^15^N on the isotope ratio mass-spectrometer as previously described.

## Results

**Figure 1:**
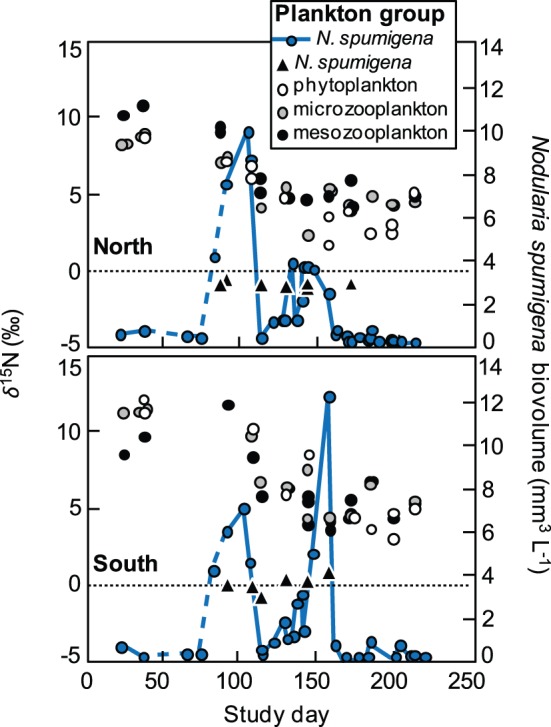
Time series of plankton and *Nodularia spumigena δ* ^15^N isotope composition and *N. spumigena* biovolumes. Time series of size-fractioned plankton and *N. spumigena δ* ^15^N isotope (‰) values, and *N. spumigena* biovolumes (mm^3^ L^−1^; blue line with circles) collected at a north (upper panel; *N*_isotope_ = 52, *N*_biovolume_ = 30) and south (lower panel; *N*_isotope_ = 48, *N*_biovolume_ = 31) sampling site in Lake King, Australia. Dashed sections of blue lines show *N. spumigena* biovolumes averaged across several locations (study days 69 and 76: *n* = 3 locations; study day 84: *n* = 7 locations) proximal to the Lake King north (upper panel) and south (lower panel) sampling sites (values represent best estimates of local biovolumes during this interval). The x-axis shows the number of days elapsed since 1 September 2011 (study day = 1).

There was a large bloom of *N. spumigena* throughout Lake King during the summer months of November 2011 to February 2012. *N. spumigena* was present in low concentrations (c. 0.5 mm^3^ L^−1^) at both sites in Lake King by late September (study day = 21) but substantial biovolumes did not develop until mid-November (study day = 89; Fig. 1). The bloom had two distinct peaks in biomass at both sites, with maximum initial and secondary biovolumes of 9.9 mm^3^ L^−1^ and 3.8 mm^3^ L^−1^ at the northern site and 7.0 mm^3^ L^−1^ and 12.2 mm^3^ L^−1^ at the southern site. By early February (study day = 155), the secondary bloom of *N. spumigena* had collapsed at both sites and biovolumes declined to concentrations of ∼ 0.2 mm^3^ L^−1^; by May *N. spumigena* was no longer detectable in the water column.

Prior to the *N. spumigena* bloom, the autotrophic plankton community was dominated by the cyanobacterium *Eucapsis* sp., the diatom *Coscinodiscus* sp. and a chlorophyte *Kirchneriella* sp. Copepods, primarily adult *Sulcanus conflictus* (Calanoida) and a mixed assemblage of unidentified adult-stage calanoids and nauplii, were the most abundant zooplankton taxa during the same pre-bloom period. There was a shift in the plankton community coincident with the *N. spumigena* bloom that was marked by declines in most of the dominant pre-bloom taxa and the appearance of previously unobserved taxa. The autotrophic plankton community of the mid-summer peak bloom period was numerically dominated by *N. spumigena* with the remainder composed of a suite of diatoms (e.g., *Aulacoseira* sp., *Coscinodiscus* sp., *Melosira* sp., *Pseusdo-nitzschia* sp., *Skeletonema costatum*), dinoflagellates (e.g., *Ceratium furca*, *Dinophysis acuminata*) and a small number (i.e., 5–6% of recorded spp.) of unidentified chlorophytes. Non-*N. spumigena* phytoplankton typically contributed an average of <5% to total autotrophic cell counts during the bloom. The cyclopoid copepod *Oncaea media* replaced *S. conflictus* as the most abundant copepod species with copepod nauplii, bivalve veligers, and ciliates (e.g., *Favella* sp.) also present. Following the final collapse of the *N. spumigena* bloom, the diatoms *Pseudo-nitzschia* cf. *pungens* and *S. costatum*, dinoflagellates *Prorocentrum minimum*, *Prorocentrum lima*, and *C. furca*, and unidentified chlorophytes dominated the phytoplankton. Abundant post-bloom zooplankton included the ciliates *Favella* sp. and *Mesodinium rubrum*, several cyclopoid copepods including *O. media*, and a mixed assemblage of copepod nauplii.

**Figure 2:**
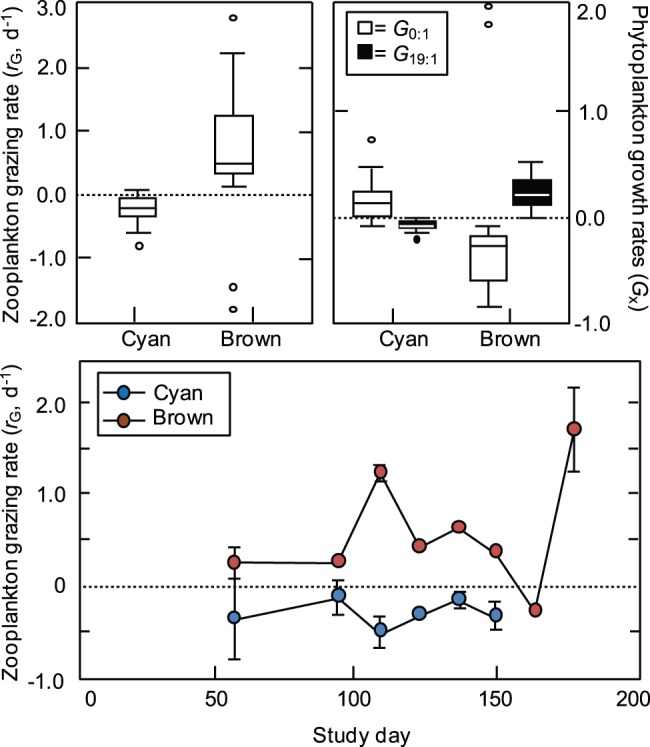
Plankton grazing and growth experiments. Zooplankton grazing rates (*r*_G_, day^−1^; upper left panel) and phytoplankton specific growth rates under different dilution conditions (*G*_0∶1_–0∶1 [filtered:ambient dilution], *G*_19∶1_–19∶1 [filtered:ambient]; upper right panel) calculated from mesocosm experiments with subsurface water from Lake King, Australia. Abscissae of upper plots coded by fluorescence color channels: cyan (cyanobacteria), brown (diatoms/dinoflagellates). Box plot components: measure of central tendency = median, outer edges of box = 25^th^ and 75^th^ interquartile range, whiskers = quartiles –(1.5×interquartile range), circles = outliers. Time-series of average *r*_G_ (error bars show ±1 SD) for the cyan and brown fluorescence channels (lower panel). The x-axis shows the number of days elapsed since 1 September 2011 (study day = 1).

Results from the grazing experiments indicated significant differences in the timing and magnitude of grazing rates, *r_G_* (d^−1^), between *N*. *spumigena* and a combined diatom and dinoflagellate group. For *N. spumigena*, growth rate peaked at *G* = 0.34±0.12 (SD) d^−1^ on study day = 106, coincident with the first peak in bloom biovolume. Among the diatoms and dinoflagellates, *G* peaked later in the time series at 0.46±0.15 on study day = 161 following the collapse of the *N. spumigena* bloom. Estimates of specific grazing rates (Fig. 2) indicated that grazers avoided *N. spumigena* (*r_G_* = −0.25±0.23) and preferentially selected (separate variance *t*-test; *t* = −4.20, *df* = 26.2, *p*<0.001) the diatoms and dinoflagellates (*r_G_* = 0.62±0.97). These taxon-specific patterns in grazing rate were consistent over the course of the bloom cycle (Fig. 2). The negative *r_G_* observed for *N. spumigena* was due to increased growth rates occurring at higher grazer concentrations, suggesting that increased grazing pressure on sympatric diatoms and dinoflagellates reduces nutrient limitation for the ungrazed *N. spumigena* [11].

Plankton isotope dynamics followed very similar trends between the two sampling locations (Fig. 1). Throughout the bloom, *δ* ^15^N values of the *N. spumigena* were consistently near or below zero at −0.9±0.1 (SD) ‰ and −0.1±0.5 ‰ for the northern and southern site, respectively. Negative *δ* ^15^N values in this range of are typical of nitrogen-fixing cyanobacteria such as *N. spumigena* [31]. Relative to *N. spumigena*, *δ* ^15^N values of the other plankton groups were enriched, with pre-bloom isotope values spanning 8.8–11.8 ‰ (Fig. 1). Following the onset of the bloom, *δ* ^15^N values of non-*N. spumigena* plankton groups declined by an average of 5.6 ‰ over the course of three months to 4.4±0.3 ‰ in the 0.07–63 µm size class (hereafter “phytoplankton”), 3.9±1.8 ‰ in the 80–149 µm size class (hereafter “microzooplankton”) and 4.9±0.7 ‰ in the ≥150 µm size class (hereafter “mesozooplankton”) by early February. During the bloom, daily rates of change in *δ* ^15^N among plankton groups ranged from −0.03 to −0.07 ‰ d^−1^ and were consistent within size classes between sites.

**Figure 3:**
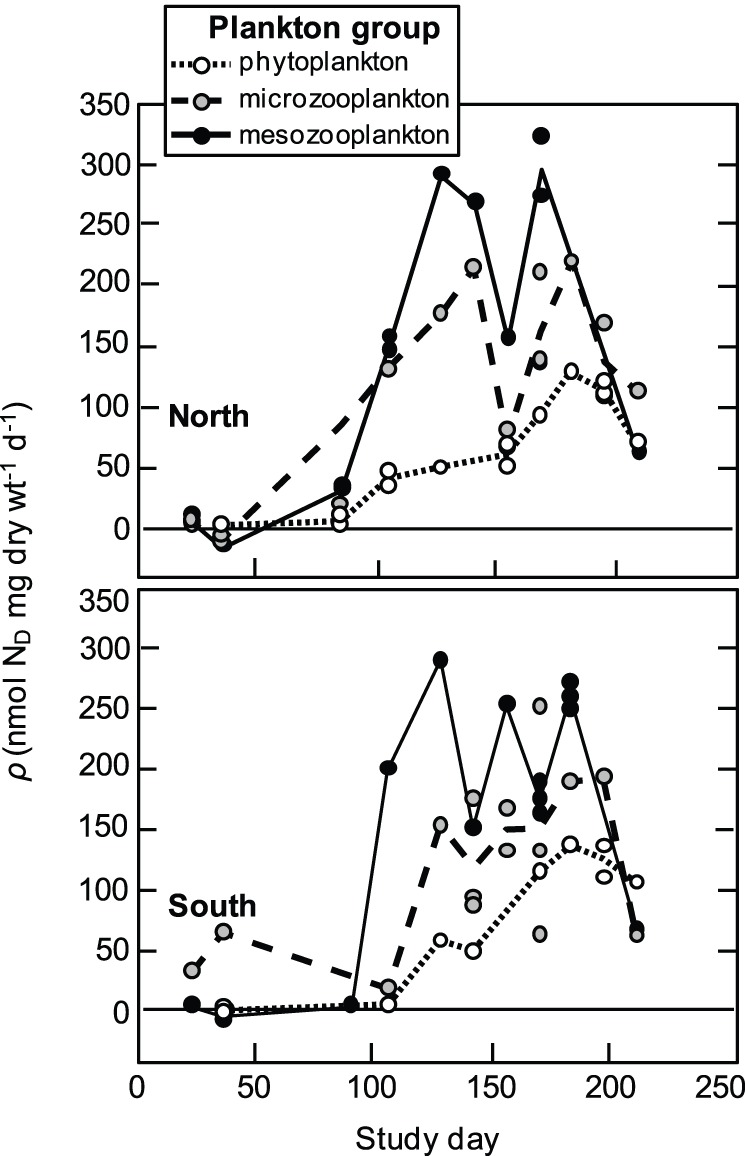
Time series of plankton diazotrophic nitrogen flux rates. Time series of size-fractioned plankton N_D_ flux rates (nmol N_D_ mg dry wt^−1^ day^−1^) collected at a north (upper panel; *N*_isotope_ = 52) and south (lower panel; *N*_isotope_ = 48) sampling site in Lake King, Australia. The x-axis shows the number of days elapsed since 1 September 2011 (study day = 1).

Estimates of mass-specific N_D_ flux *ρ* (µmol diazotrophic N mg dry wt^−1^ day^−1^) into plankton groups indicated rapid and dynamic transfer of N_D_ into all measured compartments of the planktonic food web (Fig. 3). Least-squares linear regression of *ρ* over time in the phytoplankton showed N_D_-flux accelerated consistently throughout the bloom and most of the post-bloom periods (study days 89–196) at rates of 0.97±0.08 (SE) nmol N_D_ mg^−1^ d^−2^ at the north site (n = 11, *r* ^2^ = 0.90, *ρ* = 0.97×day –74.52) and 1.32±0.20 nmol N_D_ mg^−1^ d^−2^ at the south site (n = 7, *r* ^2^ = 0.89, *ρ* = 1.32×day –124.90). Mean flux rates of N_D_ into the phytoplankton peaked at *ρ* = 126.8 and 135.6 nmol N mg^−1^ d^−1^ at the north and south sites respectively, approximately 20 to 30 d after the collapse of the secondary bloom. Flux rates had declined to 68.0 (north site) and 104.8 nmol N_D_ mg^−1^ d^−1^ (south site) by the end of the time-series. Flux into the micro- and mesozooplankton groups differed from that into the phytoplankton in both the timing and magnitude of peak uptake rates (Fig. 3). Unlike the phytoplankton, *ρ* for the zooplankton groups did not increase consistently through time, but instead roughly mirrored *N. spumigena* biovolumes at a temporal lag of 23±13 (SD) d (microzooplankton) and 21±11 d (mesozooplankton). Mean peak *ρ* values for the microzooplankton ranged from 211.4–217.1 nmol N_D_ mg^−1^ d^−1^ at the north site and 151.8–191.8 nmol N_D_ mg^−1^ d^−1^ at the south site. Mesozooplankton displayed comparable uptake maxima, with *ρ* values of 288.7–295.0 nmol N_D_ mg^−1^ d^−1^ at the north site and 288.4–258.9 nmol N_D_ mg^−1^ d^−1^ at the south site. Similar to the phytoplankton, *ρ* had declined by the end of the time-series at both sites to 60.5–109.8 nmol N_D_ mg^−1^ d^−1^ in the microzooplankton and 60.4–65.8 nmol N_D_ mg^−1^ d^−1^ in the mesozooplankton.

**Figure 4:**
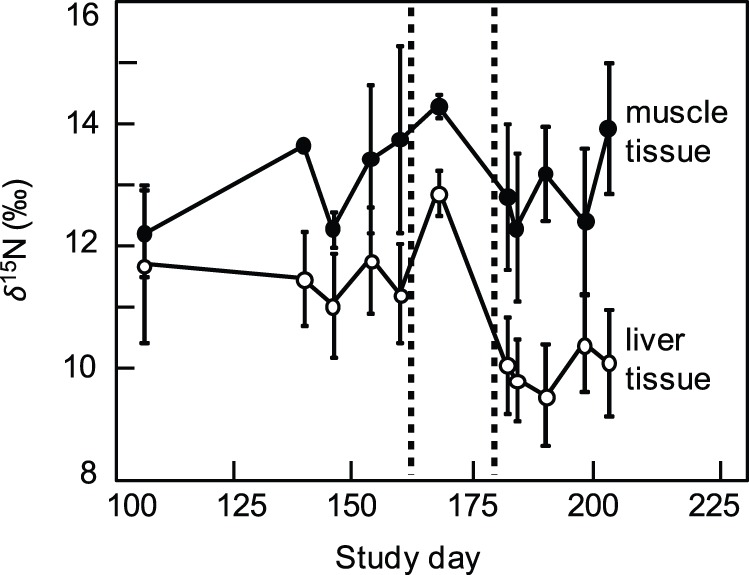
Time series of black bream muscle and liver δ^15^N isotope composition. Time series of mean *δ* ^15^N isotope (‰; error bars show ±1 SD) values of black bream *Acanthopagrus butcheri* liver (empty circles) and white muscle tissue (filled circles) collected in Lake King, Australia. Area bounded by vertical dotted lines indicate the intervening period between *N. spumigena* bloom collapse (study day ∼ 155) and depletion of liver *δ* ^15^N values. The x-axis shows the number of days elapsed since 1 September 2011 (study day = 1).

Tissue-specific *δ* ^15^N values for black bream displayed different patterns over the course of the time-series (Fig. 4). White muscle composition was consistent throughout the bloom and post-bloom period, ranging from 12.1±0.7 (SD) ‰ on study day 106 to 13.9±1.1 ‰ by study day 202. Conversely, *δ* ^15^N values of liver tissue underwent rapid depletion approximately 2–4 weeks post-bloom, declining from an average value of 11.3±0.9 ‰ during the bloom period (study days: 106–159) to 9.9±0.1 ‰ after the post-bloom depletion (study days: 181–202). Post-hoc tests of differences between bloom and post-bloom periods for each tissue showed no difference for muscle tissue (n = 31, *t* = 0.49, *p* = 0.63); whereas, the decline in liver *δ* ^15^N was significant between bloom periods (n = 31, *t* = 4.94, *p*<0.001; see Fig. 4).

## Discussion

Using a naturally occurring stable isotope tracer, we documented the *in situ* propagation of N_D_ through multiple components of an estuarine food web. These findings unequivocally demonstrate the propagation of N_D_ from *N. spumigena* into a pelagic food web and a demersal fish species under natural conditions. These results shed light on the specific trophic mechanisms underlying the transfer of N_D_ to consumer zooplankton as well as the temporal dynamics of these mechanisms relative to the trajectory of the cyanobacterial bloom.

Within the phytoplankton, the net result of disparate dissolved nitrogen-generating pathways dominating at different bloom-stages appears to be a continuous increase in the pool of bioavailable N_D_ that peaks several weeks post-bloom. During *N. spumigena* blooms, proliferation of filamentous cells leads to the formation of tangled colonies that remain buoyant, even after senescence, due to intercellular gas-filled vesicles [9]. This buoyancy effectively nullifies sedimentation by the majority of the bloom biomass and compartmentalizes initial nutrient exchange from *N. spumigena* within the pelagic food web [32]. Both free-floating filaments and aggregate colonies of *N. spumigena* support diverse consortia of bacteria [33] that are capable of remineralizing DON exudates from growing and senescing cyanobacterial cells [18], [34]. During exponential and stationary phases of *N. spumigena* growth, the availability of N_D_ to other phytoplankton is limited to assimilation of DIN [14], [16] and DON [35], [36], [37] exudates from viable *N. spumigena*, or assimilation of remineralized organic N_D_ [16], [38]. Direct measurement of total DIN indicated that concentrations were low at both sampling sites (See Methods: *Flux rate calculations*) and nutrient addition experiments showed N-limitation among diatoms and dinoflagellates (See Appendix S1). With the onset of senescence (c. study day 155), concentrations of DIN increased at both sites at a rate of 0.45 µM day^−1^ to an average of 11.5±4.4 µM by study day 182. This increase corresponded with peak flux rates of N_D_ into the phytoplankton, presumably concurrent with maximum respiration and remineralization rates of detrital *N. spumigena* biomass [38]. Our results show that the integrative effect of these disparate pathways is to dampen the temporal variability in bioavailable N_D_ and decouple N_D_ uptake rates by phytoplankton from the short-term stochasticity of *N. spumigena* bloom dynamics.

Among the heterotrophic zooplankton, potential pathways for the transfer of N_D_ from *N. spumigena* are either via direct ingestion of *N. spumigena* cellular material or the indirect transfer of labile N_D_ through one or more intermediary functional groups. Results from our grazing experiments verified the absence of measurable grazing of *N. spumigena* by the natural zooplankton community throughout the bloom period and agree with previous work demonstrating preferential avoidance of *N. spumigena* by zooplankton grazers in the Gippsland Lakes [11] and other coastal ecosystems [9].

In the absence of direct grazing, we infer that N_D_ is being routed through bloom-associated bacteria as the primary, initial trophic intermediary between *N. spumigena* and heterotrophic zooplankton. This inference is consistent with recent experiments using ^15^N labelling and nanoscale secondary ion mass spectrometry (nanoSIMS) that indicate a rapid transfer of N_D_ from filamentous cyanobacteria to epiphytic bacteria (M. Kuypers, *personal communication*). Bacterivory is very common among the protozoan and certain metazoan (e.g., copepod nauplii) taxa that constitute the microzooplankton [21], [39], [40]. Foraging of microzooplankton upon *N. spumigena*-associated bacteria has been inferred from experimental mesocosms [38] and field collections [39] and represents the most plausible initial mechanism for the trophic transfer of N_D_ to the microzooplankton. In turn, mesozooplankton are effective predators of microzooplankton [38], [41] and scavengers of detrital material (e.g., Oncaea spp.; [40]) in addition to functioning as grazers of phytoplankton. The tight temporal covariation in *ρ* that we observed between the micro- and mesozooplankton suggests a close coupling between these two functional groups. Thus, nutrient transfer from *N. spumigena* to consortial bacteria followed by heterotrophic transfers from bacteria to microzooplankton to mesozooplankton represents the most likely trophic pathway supporting the flux of N_D_ into heterotrophic zooplankton taxa.

**Figure 5:**
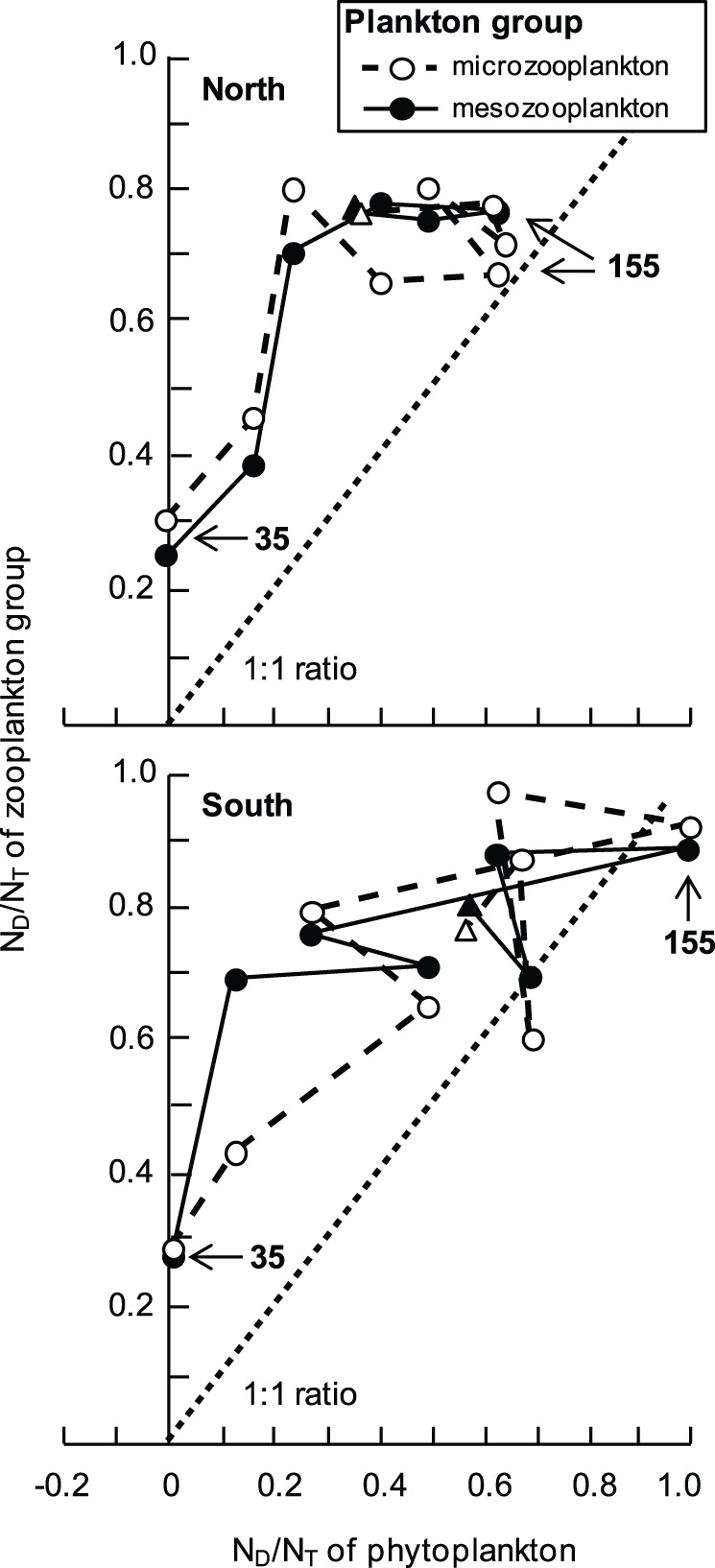
The proportional diazotrophic nitrogen content of zooplankton versus phytoplankton over time. Phase plot of the proportional contributions of diazotrophic nitrogen (N_D_) to the total nitrogen (N_T_) of microzooplanton (empty circles, final date = triangle) and mesozooplankton (filled circles, final date = triangle) versus N_D_/N_T_ of phytoplankton at the north (upper panel; *N*_microzooplankton_ = 9, *N*_mesozooplankton_ = 7) and south (lower panel; *N*_microzooplankton_ = 9, *N*_mesozooplankton_ = 8) sampling sites over the course of a *Nodularia spumigena* bloom in Lake King, Australia. Data points are daily means connected in temporal sequence by dashed (microzooplankton) or solid (mesozooplankton) lines with study day = 35 (5 October 2011) and 155 (2 February 2012; date of *N. spumigena* bloom collapse) provided for reference. The 1∶1 ratio is given by the dotted line.

Although unexpected, the higher concentration of N_D_ in the zooplankton functional groups versus the autotrophic producers throughout the bloom and immediate post-bloom phases (Fig. 5) makes sense in light of the dynamic foraging capabilities of the zooplankton taxa and compositional shifts in the dominant taxa. Mixotrophy and omnivory are common trophic strategies among micro- and mesozooplankton [40], [42] and these flexible feeding behaviours could serve to accelerate the radiation of N_D_ in these groups relative to primary producers. For example, mixotrophic dinoflagellates would have access to remineralized DIN_D_ in the water column as well as to organic N_D_ bound in the tissue of bacterial prey. Similarly, omnivorous copepod species such as *S. conflictus* would assimilate N_D_ during opportunistic grazing on diatoms or dinoflagellates, heterotrophic predation of microzooplankton [42], [43], or scavenging of biofilm-covered detritus. In particular, detrital feeding on senescent *N. spumigena* colonies could be an important mechanism by which N_D_-laden consortial bacteria and microzooplankton are consumed by mesozooplankton scavengers. Changes in the identity of dominant zooplankton taxa at different phases of the bloom could have also contributed to the rapid assimilation of N_D_. During the bloom, *S. conflictus* was replaced by the cyclopoid copepod *O. media* as the dominant mesozooplankter and several studies have documented the close association between *Oncaea* spp. and suspended detrital aggregates (i.e., marine snow; [44], [45], [46]). This association is apparently trophic – the modified maxillae of *Oncaea* spp. [45] can be used to scrape particulate-laden (<2–5 µm) biofilms from aggregate surfaces [46], [47]. Such a feeding strategy would permit direct consumption of microbial consortia associated with *N. spumigena* aggregates.

Results from the tissue analysis of black bream provide initial evidence that the post-bloom uptake of remineralized N_D_ by non-*N. spumigena* phytoplankton represents a larger fraction of the total N_D_ flux to the demersal food web and higher trophic levels. The rapid decline in black bream liver *δ* ^15^N that occurred 2–4 weeks post-bloom indicates a large pulse of isotopically light nitrogen entered the demersal food web during that time; this corresponds closely with the accelerating flux of remineralized N_D_ into the phytoplankton. Black bream are omnivores, suggesting that opportunistic foraging on available prey could explain the tight temporal coupling between N_D_ flux dynamics and this particular fish species. Together with the plankton data, these results support the hypothesis that N_D_ radiation is dominated by post-bloom processes in estuarine food webs. The observed depletion in the black bream liver *δ* ^15^N further supports the contention that remineralized bloom detritus is capable of contributing to measurable production among higher trophic levels. Interestingly, the temporal lag between the *N. spumigena* bloom cycle and the post-bloom assimilation of most N_D_ by palatable phytoplankton may interrupt the phenology of important predator-prey dynamics in the estuary. For example, the timing, magnitude and composition of spring–summer plankton cycles can be critical to the growth and survival of young-of-the-year fish and invertebrate species [48], [49]. This interval is also an important foraging period for adult-stage organisms that require sufficient energetic stores to support the metabolic demands associated with overwintering, migration or gonad development. Thus, despite the potential fertilization effect of an extended *N. spumigena* bloom, it is not clear if such a bloom represents a net positive or negative influence on productivity and food web dynamics in this (or another) estuary. Further work is needed to quantify the total flux of N_D_ during different bloom phases as well as the allocation of assimilated N_D_ to the somatic and metabolic demands of higher trophic level consumers within the estuarine food web.

**Figure 6:**
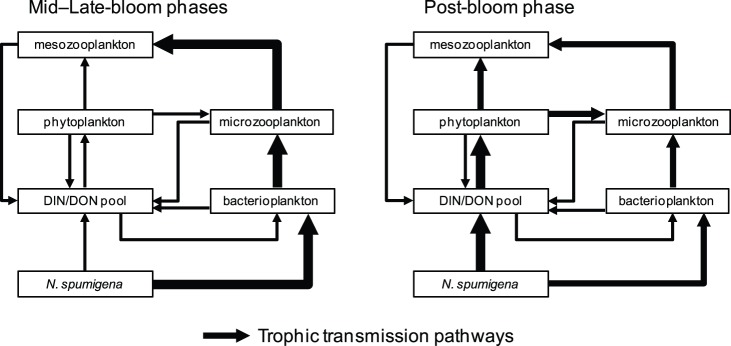
Simplified propagation pathways for diazotrophic nitrogen in a planktonic food web during different bloom phases. Simplified flow diagrams depicting differences in the primary pathways by which diazotrophic nitrogen (N_D_) was transferred from *Nodularia spumigena* to different plankton groups during (left panel) and after (right panel) a *N. spumigena* bloom in Lake King, Australia. Vectors connecting boxes indicate direction and relative magnitude of N_D_ flux through the food web. Contributions to the dissolved organic and inorganic nitrogen (DIN/DON) pool are included but have only been scaled for the relative contribution of *N. spumigena*. Plankton groups correspond with the following size-fractions: phytoplankton = 0.7–63 µm, microzooplankton = 80–149 µm, mesozooplankton ≥150 µm.

There is a growing consensus that N_D_ constitutes a potentially important subsidy for production among higher trophic levels in coastal and oceanic pelagic food webs [34], [50]. In this study, 30–95% of the phytoplankton and 55–95% of the zooplankton nitrogenous biomass was composed of N_D_ during the bloom and the post-bloom weeks (Fig. 5). In conjunction with the black bream data, this information suggests that N_D_ can rival the contribution of alternative sources of N-loading to the nutrient budget and trophic structure of estuaries during certain times of the year. Studies using a myriad of methods [34], [41], [50], [51], [52] have traced the transfer of *N. spumigena* N_D_ or biomass into different compartments of the planktonic food web; yet, translating these findings into a holistic understanding of the processes underlying the transfer of N_D_ up the food web has been hampered by the artificial conditions imposed by bench-scale experiments and (or) the short-term ‘snapshot’ characteristics of the sampling regimes. Other studies have noted the transfer of N_D_-fixation products from diazotrophic cyanobacteria to consortial bacteria [18], and have thus set the stage for our study by suggesting microbial trophic intermediaries could play an important role in the transfer of N_D_ from cyanobacteria to zooplankton. Our results provide strong support for this consensus and emphasize the trophic linkages between *N. spumigena*-associated bacterial communities and the eukaryotic elements of the planktonic food web (Fig. 6). These linkages serve to accelerate the transfer of N_D_ from *N. spumigena* into the heterotrophic components of the plankton community, leading to a tight coupling of zooplankton assimilation rates with cyanobacterial bloom dynamics. The reliance of non-*N. spumigena* phytoplankton on the dissolved pool of N_D_ and the various pathways contributing to that pool over the bloom-cycle serves to decouple phytoplankton uptake from the *N. spumigena* bloom dynamics.

## Supporting Information

Appendix S1 **Nutrient addition bioassays.** Details on methods and results from nutrient addition bioassays can be found in Appendix S1.
(DOCX)

## References

[1] Carpenter SR, Caraco NF, Correll DL, Howarth RW, Sharpley AN, et al (1998) Nonpoint pollution of surface waters with phosphorus and nitrogen. Ecol Appl 8: 559–568.

[2] Conley DJ, Paerl HW, Howarth RW, Boesch DF, Seitzinger SP, et al (2009) Controlling eutrophication: nitrogen and phosphorus. Science 323: 1014–1015. 19229022 10.1126/science.1167755

[3] Cloern JE (2001) Our evolving conceptual model of the coastal eutrophication problem. Mar Ecol Prog Ser 210: 223–253.

[4] Sharp JH, Yoshiyama K, Parker AE, Schwartz MC, Curless SE, et al (2009) A biogeochemical view of estuarine eutrophication: seasonal and spatial trends and correlations in the Delaware Estuary. Estuaries Coasts 32: 1023–1043.

[5] Anderson DM, Glibert PM, Burkholder JM (2002) Harmful algal blooms and eutrophication: Nutrient sources, composition, and consequences. Estuaries 25: 704–726.

[6] Vahtera E, Conley DJ, Gustafsson BG, Kuosa H, Pitkanen H, et al (2007) Internal ecosystem feedbacks enhance nitrogen-fixing cyanobacteria blooms and complicate management in the Baltic Sea. Ambio 36: 186–194. 17520933 10.1579/0044-7447(2007)36[186:iefenc]2.0.co;2

[7] Schindler DW, Hecky RE, Findlay DL, Stainton MP, Parker BR, et al (2008) Eutrophication of lakes cannot be controlled by reducing nitrogen input: Results of a 37-year whole-ecosystem experiment. Proceedings of the National Academy of Sciences of the United States of America 105: 11254–11258. 18667696 10.1073/pnas.0805108105 PMC2491484

[8] Cook PLM, Holland DP, Longmore AR (2010) Effect of a flood event on the dynamics of phytoplankton and biogeochemistry in a large temperate Australian lagoon. Limnol Oceanogr 55: 1123–1133.

[9] Sellner KG (1997) Physiology, ecology, and toxic properties of marine cyanobacteria blooms. Limnol Oceanogr 42: 1089–1104.

[10] Paerl HW, Fulton III RS (2006) Ecology of harmful cyanobacteria. In: Graneli E, Turner JT, editors. Ecology of Harmful Algae. Berlin-Heidelberg: Springer-Verlag. 95–109.

[11] Holland DP, van Erp I, Beardall J, Cook PLM (2012) Environmental controls on the nitrogen-fixing cyanobacterium *Nodularia spumigena* Mertens in a temperate lagoon system in South-Eastern Australia. Mar Ecol Prog Ser 461: 47–57.

[12] Rolff C (2000) Seasonal variation in δ^13^C and δ^15^N of size-fractionated plankton at a coastal station in the northern Baltic proper. Mar Ecol Prog Ser 203: 47–65.

[13] Estep MLF, Vigg S (1985) Stable carbon and nitrogen isotope tracers of trophic dynamics in natural populations and fisheries of the Lahontan Lake system, Nevada. Can J Fish Aquat Sci 42: 1712–1719.

[14] Ploug H, Adam B, Musat N, Kalvelage T, Lavik G, et al (2011) Carbon, nitrogen and O_2_ fluxes associated with the cyanobacterium *Nodularia spumigena* in the Baltic Sea. Isme Journal 5: 1549–1558. 21390075 10.1038/ismej.2011.20 PMC3160678

[15] Ploug H, Musat N, Adam B, Moraru CL, Lavik G, et al (2010) Carbon and nitrogen fluxes associated with the cyanobacterium *Aphanizomenon* sp. in the Baltic Sea. Isme Journal 4: 1215–1223. 20428225 10.1038/ismej.2010.53

[16] Bronk DA, Glibert PM (1993) Contrasting patterns of dissolved organic nitrogen release by two size fractions of estuarine plankton during a period of rapid NH_4_ ^+^ consumption and NO_2_ ^−^ production. Mar Ecol Prog Ser 96: 291–299.

[17] Paerl HW, Bebout BM, Prufert LE (1989) Bacterial associations with marine *Oscillatoria* sp (*Trichodesmium* sp) populations: Ecophysiological implications. J Phycol 25: 773–784.

[18] Paerl HW (1984) Transfer of N_2_ and CO_2_ fixation products from *Anabaena Oscillarioides* to associated bacteria during inorganic carbon sufficiency and deficiency. J Phycol 20: 600–608.

[19] Sellner KG, Olson MM, Kononen K (1994) Copepod grazing in a summer cyanobacteria bloom in the Gulf of Finland. Hydrobiologia 293: 249–254.

[20] Landry MR, Hassett RP (1982) Estimating the grazing impact of marine micro-zooplankton. Mar Biol 67: 283–288.

[21] Hambright KD, Zohary T, Gude H (2007) Microzooplankton dominate carbon flow and nutrient cycling in a warm subtropical freshwater lake. Limnol Oceanogr 52: 1018–1025.

[22] Montoya JP, Voss M, Kahler P, Capone DG (1996) A simple, high-precision, high-sensitivity tracer assay for N_2_ fixation. Appl Environ Microbiol 62: 986–993. 16535283 10.1128/aem.62.3.986-993.1996 PMC1388808

[23] Cabana G, Rasmussen JB (1994) Modeling food chain structure and contaminant bioaccumulation using stable nitrogen isotopes. Nature 372: 255–257.

[24] Post DM (2002) Using stable isotopes to estimate trophic position: models, methods, and assumptions. Ecology 83: 703–718.

[25] Vander Zanden MJ, Rasmussen JB (1999) Primary consumer δ^13^C and δ^15^N and the trophic position of aquatic consumers. Ecology 80: 1395–1404.

[26] Gu BH, Schelske CL, Brenner M (1996) Relationship between sediment and plankton isotope ratios δ^13^C and δ^15^N and primary productivity in Florida lakes. Can J Fish Aquat Sci 53: 875–883.

[27] Montoya JP, Horrigan SG, Mccarthy JJ (1990) Natural abundance of ^15^N in particulate nitrogen and zooplankton in the Chesapeake Bay. Mar Ecol Prog Ser 65: 35–61.

[28] Horrigan SG, Montoya JP, Nevins JL, Mccarthy JJ (1990) Natural isotopic composition of dissolved inorganic nitrogen in the Chesapeake Bay. Estuarine Coastal and Shelf Science 30: 393–410.

[29] Horrigan SG, Montoya JP, Nevins JL, Mccarthy JJ, Ducklow H, et al (1990) Nitrogenous nutrient transformations in the spring and fall in the Chesapeake Bay. Estuarine Coastal and Shelf Science 30: 369–391.

[30] Buchheister A, Latour RJ (2010) Turnover and fractionation of carbon and nitrogen stable isotopes in tissues of a migratory coastal predator, summer flounder (*Paralichthys dentatus*). Can J Fish Aquat Sci 67: 445–461.

[31] Bauersachs T, Schouten S, Compaoré J, Wollenzien U, Stal LJ, et al (2009) Nitrogen isotopic fractionation associated with growth on dinitrogen gas and nitrate by cyanobacteria. Limnol Oceanogr 54: 1403–1411.

[32] Heiskanen AS, Kononen K (1994) Sedimentation of vernal and late summer phytoplankton communities in the coastal Baltic Sea. Archiv Fur Hydrobiologie 131: 175–198.

[33] Tuomainen J, Hietanen S, Kuparinen J, Martikainen PJ, Servomaa K (2006) Community structure of the bacteria associated with *Nodularia* sp. (cyanobacteria) aggregates in the Baltic Sea. Microb Ecol 52: 513–522. 16944338 10.1007/s00248-006-9130-0

[34] Ohlendieck U, Stuhr A, Siegmund H (2000) Nitrogen fixation by diazotrophic cyanobacteria in the Baltic Sea and transfer of the newly fixed nitrogen to picoplankton organisms. J Mar Syst 25: 213–219.

[35] Veuger B, Middelburg JJ, Boschker HTS, Nieuwenhuize J, van Rijswijk P, et al (2004) Microbial uptake of dissolved organic and inorganic nitrogen in Randers Fjord. Estuarine Coastal and Shelf Science 61: 507–515.

[36] Bronk DA, Glibert PM (1993) Application of a ^15^N tracer method to the study of dissolved organic nitrogen uptake during spring and summer in Chesapeake Bay. Mar Biol 115: 501–508.

[37] Wawrik B, Callaghan AV, Bronk DA (2009) Use of inorganic and organic nitrogen by *Synechococcus* spp. and diatoms on the West Florida shelf as measured using stable isotope probing. Appl Environ Microbiol 75: 6662–6670. 19734334 10.1128/AEM.01002-09 PMC2772426

[38] Engström-Ost J, Koski M, Schmidt K, Viitasalo M, Jonasdottir SH, et al (2002) Effects of toxic cyanobacteria on plankton assemblage: community development during decay of Nodularia spumigena. Mar Ecol Prog Ser 232: 1–14.

[39] Gast V (1985) Bacteria as a food source for microzooplankton in the Schlei Fjord and Baltic Sea with special reference to ciliates. Mar Ecol Prog Ser 22: 107–120.

[40] Turner JT (2004) The importance of small planktonic copepods and their roles in pelagic marine food webs. Zool Stud 43: 255–266.

[41] Peters J, Renz J, van Beusekom J, Boersma M, Hagen W (2006) Trophodynamics and seasonal cycle of the copepod *Pseudocalanus acuspes* in the Central Baltic Sea (Bornholm Basin): evidence from lipid composition. Mar Biol 149: 1417–1429.

[42] Kleppel GS (1993) On the diets of calanoid copepods. Mar Ecol Prog Ser 99: 183–195.

[43] Sopanen S, Uronen P, Kuuppo P, Svensen C, Ruhl A, et al (2009) Transfer of nodularin to the copepod Eurytemora affinis through the microbial food web. Aquat Microb Ecol 55: 115–130.

[44] Green EP, Dagg MJ (1997) Mesozooplankton associations with medium to large marine snow aggregates in the northern Gulf of Mexico. J Plankton Res 19: 435–447.

[45] Ohtsuka S, Böttger-Schnack R, Okada M, Onbé T (1996) In situ feeding habits of Oncaea (Copepoda: Poecilostomatoida) from the upper 250 m of the central Red Sea, with special reference to consumption of appendicularian houses. Bulletin of Plankton Society of Japan 43: 89–105.

[46] Alldredge A (1972) Abandoned larvacean houses - a unique food source in pelagic environment. Science 177: 885–887. 17780989 10.1126/science.177.4052.885

[47] Turner JT (1986) Zooplankton feeding ecology - contents of fecal pellets of the cyclopoid copepods *Oncaea venusta*, *Corycaeus amazonicus*, *Oithona plumifera*, and *O. simplex* from the Northern Gulf of Mexico. Marine Ecology-Pubblicazioni Della Stazione Zoologica Di Napoli I 7: 289–302.

[48] Townsend DW, Cammen LM (1988) Potenial importance of the timing of spring plankton blooms to benthic-pelagic coupling and recruitment of juvenile demersal fishes. Biological Oceanography 5: 215–229.

[49] Toupoint N, Gilmore-Solomon L, Bourque F, Myrand B, Pernet F, et al (2012) Match/mismatch between the Mytilus edulis larval supply and seston quality: effect on recruitment. Ecology 93: 1922–1934. 22928420 10.1890/11-1292.1

[50] Landrum JP, Altabet MA, Montoya JP (2011) Basin-scale distributions of stable nitrogen isotopes in the subtropical North Atlantic Ocean: Contribution of diazotroph nitrogen to particulate organic matter and mesozooplankton. Deep-Sea Research Part I-Oceanographic Research Papers 58: 615–625.

[51] Loick-Wilde N, Dutz J, Miltner A, Gehre M, Montoya JP, et al (2012) Incorporation of nitrogen from N-2 fixation into amino acids of zooplankton. Limnol Oceanogr 57: 199–210.

[52] Gorokhova E (2009) Toxic cyanobacteria Nodularia spumigena in the diet of Baltic mysids: Evidence from molecular diet analysis. Harmful Algae 8: 264–272.

