# Supplementary material for: Assimilation of Diazotrophic Nitrogen into Pelagic Food Webs
Source: PLoS One. 2013 Jun 28;8(6):e67588. doi: 10.1371/journal.pone.0067588 (PMC3695911; doi:10.1371/journal.pone.0067588)
Supplement: Appendix S1 — Nutrient addition bioassays. Details on methods and results from nutrient addition bioassays can be found in Appendix S1. (DOCX) [file pone.0067588.s001.docx]

**Appendix S1** *Nutrient addition bioassays*

Nutrient addition bioassays were conducted to quantify the growth response of different components of the autotrophic community to nutrient amendment. For each iteration of the bioassays (*N* = 9), ambient water samples were collected from the northern sampling site in opaque 5-L containers for transfer back to the laboratory. Here and in later descriptions, all samples were collected from c. 20 to 25-cm subsurface to avoid potential biases associated with the unique composition of matter in surface scums.

In the laboratory, three replicate 100-ml incubations were prepared in 150-ml chambers by adding 10-µM phosphate (P) or 100-µM ammonium (N) solutions to ambient water. Replicated ambient controls were maintained for all iterations of the bioassay experiment. Incubation chambers were maintained in a water bath at *in situ* temperatures under natural light conditions: ~ 100 µmol photons m^-2^ s^-1^ illumination and a 14-hr light:10-hr dark cycle. The response of different components of the autotrophic community to nutrient amendments was analysed non-destructively in a Phytopam Phytoplankton Analyzer (Heinz Walz, GMBH, Germany). See Methods section in the primary manuscript for a full description of the method used to quantify relative fluorescence of *Nodularia spumigena* and a combined diatom-dinoflagellate functional group. Incubations were terminated after growth stabilized or reversed (7–12 d) to minimize the influence of bottle-effects on rate measurements. Data from the nutrient addition bioassays were analysed as the natural log-transformed ratio of the maximum fluorescence in the treatment bioassay to the maximum fluorescence in the control; ln*RR*. Previous work has shown that *N. spumigena* is P-limited while diatoms and dinoflagellates are N-limited [1]; therefore, we used one-tailed *t*-tests of the ln*RR* were used verify similar patterns during the 2011-2012 bloom.

Results indicated that *N*. *spumigena* experienced maximum growth enhancement in P-amended treatments: P-treatment ln*RR* = 0.64 ± 0.59 (SD), N-treatment ln*RR* = 0.07 ± 0.32 (one-tailed *t*-test: *t* = 1.96, *df* = 5, *p* = 0.05)*.* Conversely, the diatom- dinoflagellate group experienced maximum growth enhancement in N-amended treatments: P-treatment ln*RR* = 0.04 ± 0.19, N-treatment ln*RR* = 0.57 ± 0.47 (one-tailed *t*-test: *t* = -3.21, *df* = 8, *p* = 0.006). These results indicate that *N. spumigena* was P-limited throughout the bloom and, conversely, diatoms and dinoflagellates were N-limited during the growth and stationary bloom-phases.

**Works Cited**

1. Holland DP, van Erp I, Beardall J, Cook PLM (2012) Environmental controls on the nitrogen-fixing cyanobacterium *Nodularia spumigena* Mertens in a temperate lagoon system in South-Eastern Australia. Mar Ecol Prog Ser 461: 47-57.
